# Census of the fruit and flower chafers (Coleoptera, Scarabaeidae, Cetoniinae) of the Macau SAR, China

**DOI:** 10.3897/zookeys.1026.60036

**Published:** 2021-03-25

**Authors:** Renzo Perissinotto, Lynette Clennell

**Affiliations:** 1 Institute for Coastal & Marine Research (CMR), Nelson Mandela University, P.O. Box 77000, Port Elizabeth 6031, South Africa Nelson Mandela University Port Elizabeth South Africa; 2 Macau Anglican College, 109–117 Avenida Padre Tomas Pereira, Taipa, Macau SAR, China Macau Anglican College Macau Macau

**Keywords:** Cetoniini, new records, Palearctic Region, Schizorhinini, Taenioderini

## Abstract

The coleopteran fauna of the Macau SAR in southern China has historically received only limited attention and no updated information has been published since the last substantial works produced in the 1990s. An annotated and illustrated review of the fruit and flower chafers (Scarabaeidae, Cetoniinae) of this region is here presented, in order to provide an account of the current status of the taxonomic diversity and ecology of this important insect group. Eleven species were observed in the SAR during an intense investigation undertaken during the period 2017–2020, with six of these representing new records for Macau and two for the broader region of the Pearl River Delta, also known as the Greater Bay Area. Although this census leads to a substantial increase in the number of species known for the area, it also highlights the threats that the recent escalation in urban development and land-use transformation are posing to a number of species which seem unable to maintain a sustainable population in the region, mainly due to habitat destruction.

## Introduction

Fruit and flower chafers are good indicators of environmental health status and are increasingly used in environmental assessment and planning studies (e.g., Mawdsley et al. 2011; Mudge et al. 2012; Touroult and Le Gall 2013; Correa et al. 2019). They constitute a very diverse group of insects, with currently almost 5000 described species, and play a very important ecological role in processes such as pollination and nutrient recycling in the soil (Beutel and Leschen 2005). Their larvae are typical white grubs, generally living within the soil as plant detritivores or in decomposing wood as part of a saproxylic community (Correa et al. 2019). Conversely, the adults are normally brightly-coloured beetles with diurnal activity and feed on a variety of flowers, overripe fruits and tree sap flows (Beutel and Leschen 2005; Krikken 1984).

Unlike in the other Chinese SAR in the same region, Hong Kong, in Macau there has been relatively little research undertaken in the past on its insect fauna, with only a handful of publications produced on the subject to date. Among these, to our knowledge only three have reported records of occurrence of Cetoniinae, namely Easton (1991: “*Agestrata orichalea* and *Protaetia orientalis* G. & P.”), Easton (1993: “*Protaetia orientalis* Gory & Percheron”) and Pun and Batalha [1997: “*Agestrata orichalcea* Linnaeus, *Protaetia orientalis* Gory & Percheron, *Oxycetonia jucunda* Faldermann and *Thaumastopeus nigritus* (Frohlich)”]. Thus, in essence only four species in this beetle group have been reported in the literature to date. So, it is not surprising that Cetoniinae records for Macau have largely been ignored or omitted in all the major works undertaken on this group of insects in the broader Chinese region and the world. For instance, despite explicitly mentioning Macau/Macao as part of the region included in their revisions, neither Krajcik (2011) nor Bezděk (2016) mention any specific Cetoniinae record from this SAR in their reviews. Similarly, Macau does not feature at all in Sakai and Nagai’s (2008) outstanding overview of the cetoniines of the world, neither in the list of specific records, nor in any citation of geographic distribution.

This under-reporting is further compounded by issues of outdated or incorrect identifications. The main purpose of this work is, therefore, to provide a modern census of the cetoniine beetles of the Macau SAR, based on extended and frequent field surveys, comprehensive observation gathering methods and updated identification approaches using local and global expertise. To our knowledge, along with a similar study undertaken recently by Leong et al. (2017) on the ants (Hymenoptera, Formicidae) of Macau, this represents the only modern census of a group of insects undertaken during the current century in this Chinese SAR. It is hoped that this will stimulate further research and interest in the region, as well as provide the local authorities with supporting information towards their ongoing environmental management and biodiversity conservation programmes.

## Materials and methods

Macau is characterised by a subtropical climate and what remains of its natural terrestrial plant assemblages includes five vegetation types, namely coniferous forest, coniferous and broad-leaved mixed forest, evergreen broad-leaved forest, evergreen and deciduous broad-leaved mixed forest and shrub (Peng et al. 2014). Although biogeographically it is part of the Palearctic Region, it lies at the interface with the Oriental Region and, consequently, there is a large overlap in the occurrence of species from both regions within its boundaries (Löbl and Löbl 2016).

**Figure 1.**
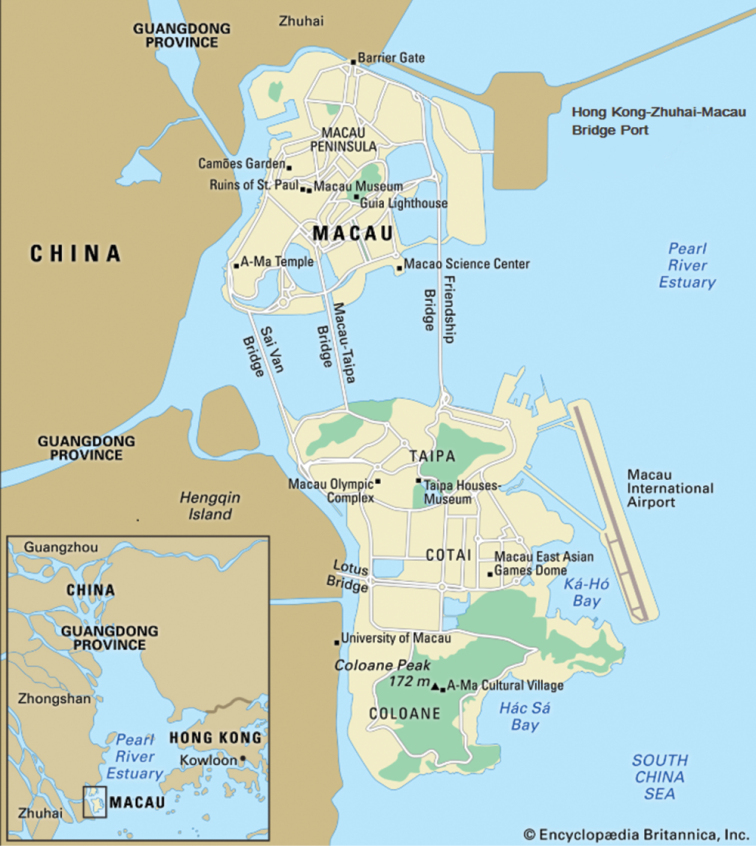
Map of the Macau SAR showing its various components including the Peninsula, the islands of Taipa and Coloane as well as the reclaimed lands of Cotai, the International Airport and the Hong Kong – Zhuhai – Macau Bridge Port (adapted from: https://www.britannica.com; used with permission).

The Macau Special Administrative Region (SAR) of China consists of the Macau Peninsula, linked directly to the mainland province of Guangdong, and one larger island resulting from the merger of the two previous islands of Taipa and Coloane through the land reclaimed area of Cotai (Fig. 1). Other land reclamations have also added the International Airport to the Taipa-Coloane complex and more recently the Hong Kong-Zhuhai-Macau Bridge Port to the Peninsula, which now connects the three regions that constitute the so-called Greater Bay Area of the Pearl River Estuary (Fig. 1). An advanced network of road and bridge infrastructure also connects all the components of the SAR, which currently has a total areal extent of ca. 30 km^2^ (Leong et al. 2017).

**Figure 2.**
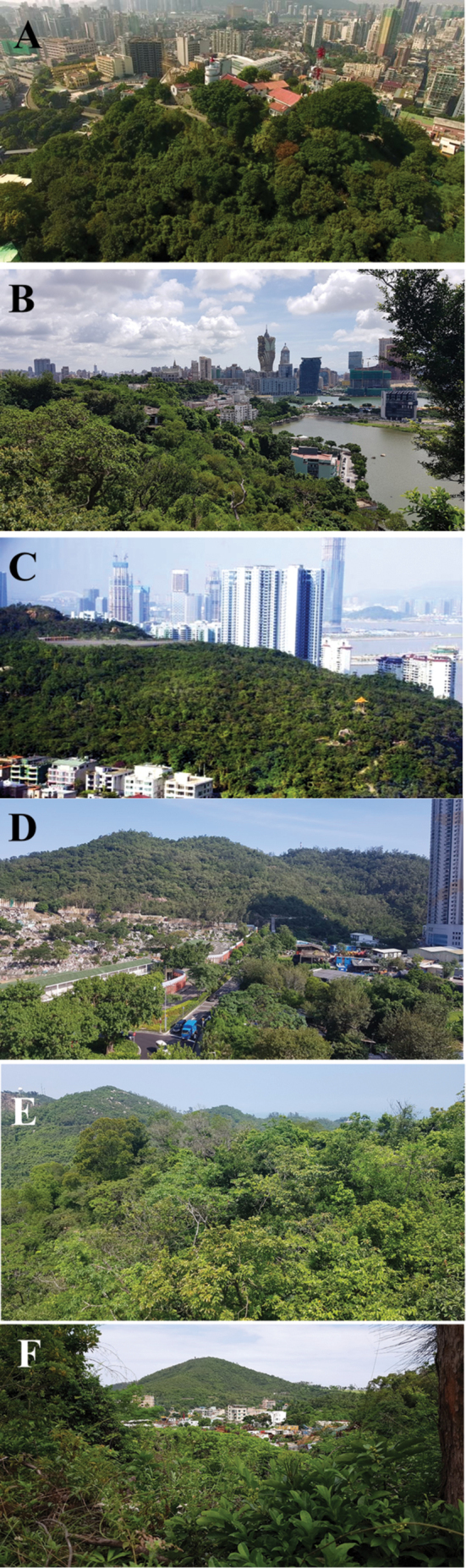
Examples of remaining pockets of subtropical evergreen forest in Macau **A** Colina da Guia (Macau Peninsula) **B** Colina da Barra (Macau Peninsula) **C** Taipa Pequena (Taipa) **D** Taipa Grande (Taipa) **E** Alto de Coloane (Coloane) **F** Monte de Ká-Hó (Coloane). Photographs: **A** Shutterstock.com **C** JTM.co.mo **B, D–F** Lynette Clennell.

The Macau SAR is a very prosperous region, reportedly enjoying one of the highest per capita incomes in the world, but is also among the most densely populated areas on the planet. Remarkably, despite its extraordinary population pressure and developmental momentum, some areas of its original, natural landscape still remain, although they are often encroached upon by alien vegetation (Leong et al. 2017). These consist mainly of densely forested hilly outcrops intersected by networks of hiking trails, service roads and recreational facilities. The largest are located in the Coloane area (e.g., Alto de Coloane, Barragem de Ká-Hó and Monte de Ká-Hó) and Taipa (Taipa Grande and Taipa Pequena), but there are lesser pockets in the Peninsula as well (e.g., Colina da Guia, Colina da Barra, Parque Municipal de Mong Há and Ilha Verde) (Figs 1, 2). All these sites were visited on a regular basis during the census period, in order to provide an areal cover as comprehensive as possible of the potential habitats for Cetoniinae within the SAR.

Cetoniine observations were undertaken on an opportunistic basis from Sep 2017 and virtually on a daily basis during the two-year period Oct 2018–Oct 2020. This generally involved non-manipulative methods, with photographs taken in situ as much as possible. Fruit-baited traps were deployed during the summer of 2019 in urban marginal areas, with the main purpose of attempting to run a mark-recapture exercise and estimate the numerical abundance for the various species. Unfortunately, too few specimens and species were found to enter the traps and therefore the attempt was abandoned thereafter. Traps were made using a standard 1 L bottle with the neck cut-off and inverted inside the bottle in order to create a funnel-like entrance that would allow beetles to enter, but not re-exit (Touroult and Le Gall 2013; Correa et al. 2019). The bait consisted of a variety of fermenting fruits, mainly banana, lychee, pineapple and grape, mixed with brown sugar and red wine. Traps were suspended on tree branches, ca. 2–3 m above the ground and inspected on a daily basis. Trapped beetles were sexed, sized, photographed and immediately released.

Occasionally, mature adults ready to emerge were excavated from decomposing tree trunks still in their cocoons, or obtained after rearing third instar larvae collected in the wild, under laboratory controlled-conditions. In the latter case, larvae were kept in plastic containers of 1 L capacity, containing the natural wood material found in situ. Water was sprayed on the soil surface at regular intervals of ca. 1–2 weeks until pupation. Voucher specimens for reference and identification verification purposes were usually selected from specimens found already dead in the field. These are currently housed in the Macau Anglican College, Taipa (**MACT**) or in the reference collection of Stanislav Jákl, Prague (**SJCP**) for further investigation. Other specimens for analysis were accessed from the historical Easton Collection currently housed in the Library of the University of Macau (**UMEC**). Preserved specimens were analysed in detail for the typical diagnostic characters of each species, including aedeagal parameres. Observations and data records were also obtained from the citizen science platform iNaturalist (www.inaturalist.org), after direct verification with each individual observer. The following literature references were used to extract historical information records: Easton (1991), Easton (1993) and Pun and Batalha (1997).

Photographs of specimen dorsal and lateral views were generally taken in situ as far as practical, using a Nikon CoolPix S9700 digital camera with macro setting. Where this was not possible in the field, specimens were photographed, sexed and measured under controlled conditions and released immediately afterwards. On rare occasions, visual disturbances were removed from the photographs using Microsoft Word 2010 (Picture Tools), in order to increase clarity and resolution of the images. Specimen total length (TL) and maximum width (MW) were measured using a Vernier calliper, from the anterior margin of the clypeus to the apex of the pygidium and at the widest point of the elytra, respectively.

In this work, all the species recorded during the census in the Macau SAR are illustrated with photos of live specimens in their natural or reconstructed setting, highlighting their key dorsal and, where possible, lateral characters. For a comprehensive list of currently recognised synonyms, the reader is referred to the latest revision of the Palearctic Coleoptera by Löbl and Löbl (2016).

## Taxonomy

### Tribe Cetoniini Leach, 1815

#### Genus Gametis

Taxon classificationAnimaliaColeopteraCetoniidae

Burmeister, 1842

36ACDBAF-F536-54C2-AD0D-08838805A569

##### Type species.

*Cetonia versicolor* Fabricius, 1775.

#### Gametis bealiae

Taxon classificationAnimaliaColeopteraCetoniidae

(Gory & Percheron, 1833)

96856314-5310-59E5-937E-885D807988DA

Fig. 3

Cetonia bealiae Gory & Percheron, 1833: 282.

##### Distribution.

Within the Palearctic Region, *G. bealiae* is known from the Chinese provinces of Fujian, Guangdong, Hebei, Hubei, Jiangxi, Zhejiang and the Hong Kong SAR (Yiu 2010; Lirong et al. 2013; Bezděk 2016). It is also widely distributed across the Oriental Region, specifically in Myanmar, Laos, northern Vietnam and north-eastern India (Sakai and Nagai 1998; Krajcik 2011).

##### Material examined.

1♂: Coloane Village, 29 Jun 2019, in fruit-baited trap, R Perissinotto & L Clennell (MACT).

##### Other Macau records.

Taipa Pequena, 4 Mar 2019, on flowers of *Ligustrum sinense*, R Perissinotto; Coloane, Hác-Sá, 4 Apr 2019, on flowers of *L. sinense*, R Perissinotto & L Clennell; ibidem 11 Apr 2020, R Perissinotto & L Clennell.

##### Remarks.

In Macau, this species varies in size between 12.5 and 15 mm in TL and between 7 and 9 mm in MW. Colour forms range from black to olive green background, with testaceous to reddish green pronotum and ochreous to testaceous mid-elytral bands. The white maculation on the general surface appears to be consistent. During 2019, one individual was retrieved inside a fruit-baited trap, while four others were observed feeding on flowers of *Ligustrum sinense*. In 2020, only one specimen was observed while feeding on flowers of *L. sinense*. In Macau, this species appears to have its peak of adult activity between early spring and early summer, while no specimens have been recorded in late summer, autumn, or winter. In nearby Hong Kong, this species has been recorded feeding on flowers of *Viburnum odoratissimum* and *Lonicera* sp. between March and May (Yiu 2010) as well as on flowers of *Zanthoxylum avicennae* in October (https://www.inaturalist.org/observations/62885551). No information is available on larval or pupal stages in the region.

**Figure 3.**
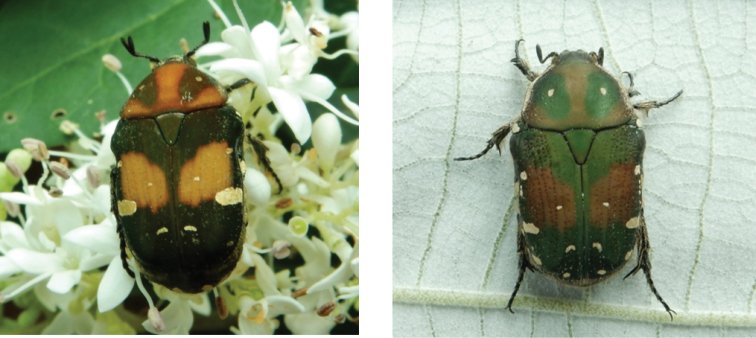
*Gametis bealiae* (Gory & Percheron, 1833): dark green and ochreous form (left), olive green and testaceous form (right) observed at Coloane on 4 Apr 2019 (photographs: Lynette Clennell).

#### Gametis jucunda

Taxon classificationAnimaliaColeopteraCetoniidae

(Faldermann, 1835)

1431720A-06FD-5F17-A9D2-C29EF3512AC2

Fig. 4

Cetonia jucunda Faldermann, 1835: 386.

##### Distribution.

Mainly a Palearctic species, recorded from the Chinese provinces and municipalities of Beijing, Chongqing, Fujian, Gansu, Guizhou, Guangxi, Hainan, Hebei, Heilongjiang, Hubei, Jiangsu, Jiangxi, Liaoning, Nei Mongol, Sichuan, Shanghai, Shandong, Shanxi, Yunnan, Zhejiang and the Hong Kong SAR (Lirong et al. 2013; Bezděk 2016). Also found in Pakistan, Nepal, the Indian Sikkim Province, North and South Korea, Mongolia, Russian Far East and Japan (Sakai and Nagai 1998; Krajcik 2011; Bezděk 2016). According to Bezděk (2016), it also occurs in the Oriental Region.

##### Material examined.

1♂, 1♀: Coloane, Hác-Sá, 8 Apr 2020, on flowers of *Ligustrum sinense*, R Perissinotto (MACT).

##### Other Macau records.

No locality and date, 14 mm (in Pun and Batalha 1997: 65, fig. 108 as *Oxycetonia jucunda* Faldermann); Taipa Pequena, 11 Mar 2019, on flowers of *Toddalia asiatica*, R Perissinotto & L Clennell.

##### Remarks.

A rare species in Macau, despite its widespread distribution and common occurrence in the surrounding regions, such as the Hong Kong SAR (Yiu 2010; iNaturalist observations). The dorsal background colour is always predominantly green, ranging from bright to olive grade, while the white maculation can vary somehow in extent. In particular, the discal spots on both pronotum and elytra can fade completely in exceptional cases. Specimens range in size within the approximate range of 12–15 mm TL and 6–8 mm MW. The period of adult activity appears to peak in the spring and no specimens have been recorded in Macau during summer or autumn yet, although their occurrence during these seasons is well established in nearby Hong Kong (Yiu 2010). In Macau, adults were observed feeding on flowers of *Ligustrum sinense*, *Clausena lansium* and *Toddalia asiatica*, while in Hong Kong they were also found on *Guilandina bonduc*, *Rhus chinensis* and *Schima superba* (Yiu 2010). The 1^st^–3^rd^ instar larvae of *G. jucunda* have been comprehensively described and illustrated by Murayama (1931), Medvedev (1952), Zhang (1984) and Sawada (1991) (Šípek and Král 2012).

**Figure 4.**
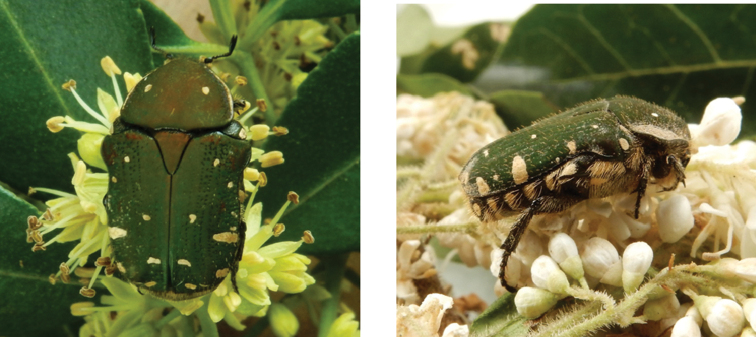
*Gametis jucunda* (Faldermann, 1835): dorsal (left) and side (right) views of typical specimens observed at Taipa (11 Mar 2019) and Coloane (8 Apr 2020), respectively (photographs: Lynette Clennell).

### Genus *Glycyphana* Burmeister, 1842

#### Subgenus: Glycyphana

Taxon classificationAnimaliaColeopteraCetoniidae

Burmeister, 1842

45A7C5B2-D95F-5688-8687-E5637CB83D3E

##### Type species.

*Cetonia horsfieldii* Hope, 1831.

#### Glycyphana (Glycyphana) horsfieldii

Taxon classificationAnimaliaColeopteraCetoniidae

Hope, 1831

BFBCBBF5-2771-5F59-B005-C6CBF791338D

Fig. 5

Cetonia horsfieldii Hope, 1831: 25.

##### Distribution.

Known in the Palearctic Region from the Himalayan countries of Nepal, Bhutan, and the Sikkim Province of India, as well as the Chinese provinces of Guizhou, Jiangxi, Yunnan, and the island of Taiwan (Bezděk 2016). It has only recently been recorded from Hong Kong for the first time (Aston and Melsom 2019). In the Oriental Region, it has been reported from Myanmar, Vietnam, Thailand, Laos, India and Sri Lanka (Sakai and Nagai 1998; Krajcik 2011).

##### Material examined.

1♂: Taipa Monument, 27 Sep 2018, dead on path, R Perissinotto (MACT); 1♀: Coloane Village, 28 Aug 2019, on flowers of *Zanthoxylum avicennae*, R Perissinotto & L Clennell (MACT).

##### Other Macau records.

Macau, Guia Hill, 25 Oct 2017, R Perissinotto & L Clennell; Taipa Pequena, 4 May 2018, R Perissinotto & L Clennell; ibidem 11 Oct 2018, R Perissinotto & L Clennell; Macau Peninsula, 22 Dec 2019, Angus Chan (pers. comm.); Coloane, Hác-Sá, 7 Apr 2020, on flowers of *Ligustrum sinense*, R Perissinotto & L Clennell.

##### Remarks.

This species appears to be a new record for the broader region, having also been observed for the first time in Hong Kong only in April 2018 (Aston and Melsom 2019). The subspecies *G. h. chinensis*, originally described by Mikšić (1970) based on its wider extent of the red pronotal margin over the nominal subspecies (e.g., Ma 1995), is no longer recognised and has been synonymised with *G. horsfieldii* (Jákl in Löbl and Löbl 2016: 18). Specimens in Macau range in size from approximately 13 to 16 mm TL and from 6 to 8 mm MW. The period of adult activity seems to extend throughout the year, but most observations are from the spring (Apr–May) and autumn (Sep–Oct) months. While the larval stages are not known, adults have been recorded on flowers of *Ligustrum sinense*, *Zanthoxylum avicennae*, *Shefflera heptaphylla* and *Homalium cochinchinense*.

**Figure 5.**
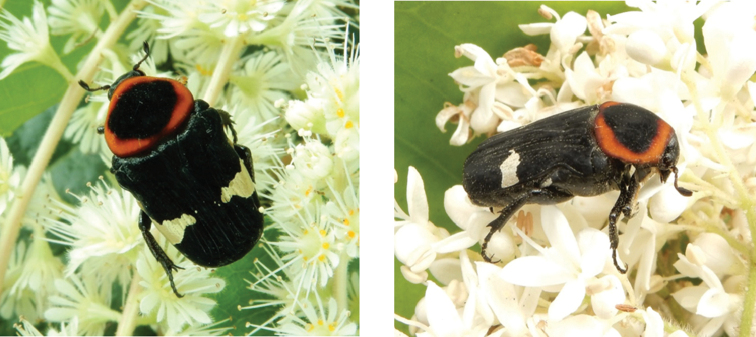
Glycyphana (Glycyphana) horsfieldii Hope, 1831: dorsal (left) and side (right) views of typical specimens observed at Taipa (11 Oct 2019) and Coloane (7 Apr 2020), respectively (photographs: Lynette Clennell).

#### Subgenus  Glycyphaniola

Taxon classificationAnimaliaColeopteraCetoniidae

Mikšić, 1968

B08623E3-6E66-56A4-8744-FA3308F7133A

##### Type species.

*Cetonia modesta* Fabricius, 1792

#### Glycyphana (Glycyphaniola) laotica

Taxon classificationAnimaliaColeopteraCetoniidae

Mikšić, 1968

1B90E994-423E-5E25-8F46-F4B780F76D20

Fig. 6

Glycyphaniola nicobarica laotica Mikšić, 1968: 48.

##### Distribution.

According to Jákl (in Löbl and Löbl 2016: 18), it is currently known from the Hong Kong SAR and parts of the Oriental Region. It is likely that it occurs through much of southern China.

##### Material examined.

1♀: Taipa Central, October 2017, Jeff Lei (MACT); 1♂: Barra Hill, 5 May 2018, dead on roadside, L Clennell (MACT); 1♂: Taipa Pequena, 26 Sep 2018, on flowers of *Rhus chinensis* by roadside, R Perissinotto & L Clennell (MACT); 1♀: Coloane Village , 13 Mar 2019, on flowers of *Bidens* sp., R Perissinotto (SJCP); 1♂: ibidem 13 Apr 2019, R Perissinotto (SJCP).

##### Other Macau records.

Taipa Pequena, 26 Oct 2017, on flowers of *Rhus chinensis*, R Perissinotto & L Clennell; ibidem 2 Mar 2020; Coloane Village , 2 Jul 2019, on flowers of *Bidens* sp., R Perissinotto & L Clennell; ibidem, 29 Sep 2018, R Perissinotto & L Clennell; Coloane, Ká-Hó, 7 Oct 2018, R Perissinotto & L Clennell; Macau, Guia Hill, 14 Mar 2020, on flowers of *Ligustrum sinense*, R Perissinotto & L Clennell; St. Francis Xavier’s Parish, Macau [Coloane], 7 Jul 2019 13:04, Kit Chang (https://www.inaturalist.org/observations/28360614); ibidem 12 Apr 2020 13:29, L Clennell (https://www.inaturalist.org/observations/55132359); ibidem 27 Sep 2020 14:59, L Clennell (https://www.inaturalist.org/observations/60940151); ibidem 30 Sep 2020, L Clennell (https://www.inaturalist.org/observations/61293565); Our Lady of Carmel’s Parish [Taipa], 17 Aug 2020 14:14, L Clennell (https://www.inaturalist.org/observations/56723739); ibidem 14 Sep 2020 16:07, Kit Chang (https://www.inaturalist.org/observations/59561942); Circuito da Barragem de Hac-Sá, Coloane, 4 Jul 2020 15:26, Annie Lao (https://www.inaturalist.org/observations/51892005).

##### Remarks.

**Figure 6.**
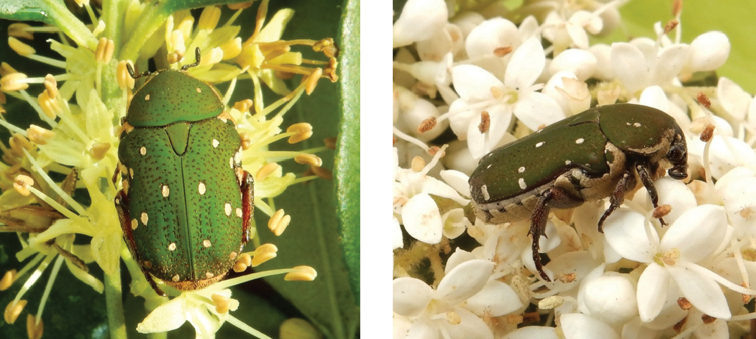
Glycyphana (Glycyphaniola) laotica Mikšić, 1968: dorsal (left) and side (right) views of typical specimens observed at Taipa (14 Mar 2019) and Coloane (20 Apr 2020), respectively (photographs: Lynette Clennell).

This taxon has recently been elevated to species rank by Jákl (in Löbl and Löbl 2016: 18), on the basis of comparative studies of numerous specimens from continental Asia and the type material of *Glycyphana nicobarica* Janson, 1877. Thus, all previous identifications of the local G. (Glycyphaniola) species occurring in Macau and nearby Hong Kong almost certainly refer to this species rather than to *G. nicobarica*, which is probably an endemic to the Nicobar Islands (S Jákl, pers. comm.). Specimens range in size between approximately 9–12 mm in TL and 5–6 mm in MW. The background colour of their body surface can vary from bright light green, to dark olive-green (Fig. 6) and in extreme cases even brown to brick-red. The extent of white maculation also varies substantially across both elytral and pronotal surfaces.

Larvae, cocoons, and freshly emerged adults have been observed inside decomposing tree trunks and branches (pers. obs.), thereby confirming the saproxylic habit of its immature stages. In Hong Kong (then referred to as *Glycyphana nicobarica*), larvae were successfully reared to adulthood on fermented sawdust, and several adult specimens were found inside a compost heap composed of pig waste and sawdust (Yiu 2010). Adults have been recorded feeding on a variety of flowers, including *Rhus chinensis*, *Ligustrum sinense*, *Mallotus paniculatus*, *Murraya paniculata*, *Schefflera heptaphylla*, *Viburnum odoratissimum*, *Zanthoxylum avicennae*, *Bauhinia championii*, and even on the alien invasive herbs *Bidens alba* and *B. pilosa*. They do not seem to be attracted to fruit-baited traps and have not been observed on sap flows either. This is one of the most often encountered species in the Macau SAR, but never in abundance. It occurs all year round, with distinct peaks of adult activity in the spring (Mar–Apr) and autumn (Sep–Oct).

#### Genus Protaetia

Taxon classificationAnimaliaColeopteraCetoniidae

Burmeister, 1842

BEABEF80-6902-51F8-BFE4-6AF6ADDA282E

##### Type species.

*Cetonia mandarina* Weber, 1801 (= *Cetonia fusca* Herbst, 1790).

#### Subgenus  Calopotosia

Taxon classificationAnimaliaColeopteraCetoniidae

Reitter, 1899

5C4E5377-BD03-5F4F-A7C5-8E4529521255

##### Type species.

*Cetonia submarmorea* Burmeister, 1842.

#### Protaetia (Calopotosia) orientalis orientalis

Taxon classificationAnimaliaColeopteraCetoniidae

(Gory & Percheron, 1833)

C4D5F2D9-35F5-5383-A13A-51D117ADDF8D

Fig. 7

Cetonia orientalis Gory & Percheron, 1833: 193

##### Distribution.

Within the Palearctic Region, this species is known from the Chinese provinces of Chongqing, Fujian, Guangxi, Guizhou, Hubei, Hunan, Jiangxi, Sichuan, Shandong, Zhenjiang, the Hong Kong SAR, North and South Korea as well as the Russian Far East (Lirong et al. 2013; Bezděk 2016). It is also widespread in the Oriental Region, specifically in northern India, the Kashmir region, northern Vietnam, and Laos (Sakai and Nagai 1998; Krajcik 2011).

##### Material examined.

1♂, 1♀: Coloane, Ká-Hó, 16 Jun 2018, dead on roadside, L Clennell (MACT); 1♂: Coloane, A-Mà Cultural Village, 13–15 Jun 2019, aggregation on sap of *Albizia lebbeck*, R Perissinotto & L Clennell (MACT); 1♂: Macau, University of East Asia Library, 4 May 1990, ER Easton leg (UMEC); 1♀: ibidem, on building, 1 Aug 1989, ER Easton leg (UMEC); 1♂: ibidem 30 Jul 1989, ER Easton leg (UMEC); 1♂: ibidem, on building, 12 Jul 1989, ER Easton leg (UMEC); 1♀: ibidem no data, ER Easton leg (UMEC).

##### Other Macau records.

Taipa, University of East Asia Campus, near library, 28 May 1991 (Easton 1991: 111; 1993: 55); No locality and date, 18 mm (Pun and Batalha 1997: 66, fig. 109); Taipa Grande, 22 May 2018, L Clennell; Coloane, 10 May 2019, R Perissinotto & L Clennell; ibidem 18 May 2019, R Perissinotto & L Clennell; Macau, 15 Jun 2019, Peggi Chao (pers. comm.); ibidem 8 Aug 2019, Ben Wong (pers. comm.); Alto de Coloane, 23 May 2020, R Perissinotto & L Clennell; ibidem 5 Jul 2020, feeding on sap of *Sapium discolor* R Perissinotto & L Clennell; Barra Hill, Macau, 9 Jul 2018 12:58, Kisu Wong (https://www.inaturalist.org/observations/23843295); Coloane Alto, Macao, 15 Jun 2019 16:11, L Clennell (https://www.inaturalist.org/observations/56299608); St. Francis Xavier’s Parish [Coloane], 12 May 2019 10:27, Kit Chang (https://www.inaturalist.org/observations/24989100); ibidem 5 Jul 2020 13:56, L Clennell (https://www.inaturalist.org/observations/56110922); Avenida Doutor Stanley Ho [Macau], 24 May 2020 11:44, Benny Kuok (https://www.inaturalist.org/observations/47261733).

##### Remarks.

**Figure 7.**
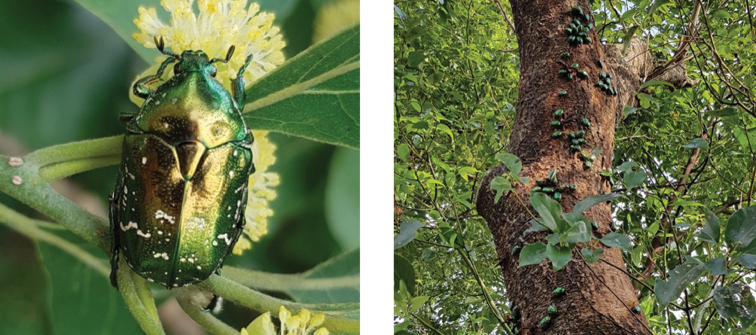
Protaetia (Calopotosia) orientalis orientalis (Gory & Percheron, 1833): dorsal view of typical specimen (left) and aggregation of several individuals (right) on sap flow of *Albizia lebbeck*, observed at Coloane on 18 May 2019 and 15 Jun 2019, respectively (photographs: Lynette Clennell).

This is the only species currently seen in reasonable numbers in the Macau SAR, but only from late spring and throughout the summer. Specimens are generally of a bright green colour with golden sheen and white markings, but the background colour can turn olive-green or even darker in some specimens. Adult size varies within the approximate range of 20–25 mm in TL and 10–13 mm in MW. Its diet appears to be the most variable exhibited by any of the cetoniines encountered in Macau, with adults recorded in aggregations on sap flows of *Albizia lebbeck* (Fig. 7) and *Sapium discolor*. It also enters regularly into fruit-baited traps and has been observed feeding on wild figs and lychee fruits. Among the flowering trees that attract this species are *Acronychia pedunculata*, *Litsea glutinosa*, *Paliurus spina-christi* and *Syzigium buxifolium*.

Easton (1993) reported that this species was regarded a minor pest, as the adults fed on lychees and other soft fruits such as peaches, while their grubs were believed to feed on the roots of grasses. Although adults are typically diurnal, with activity peaking in the hottest part of the day, they have also been found on the walls of buildings illuminated at night (Easton 1993). The 3^rd^ instar larva of this species was comprehensively described and illustrated by Sawada (1991). In Hong Kong, larvae have been successfully reared in captivity using fermented sawdust as food (Yiu 2010).

#### Subgenus Liocola

Taxon classificationAnimaliaColeopteraCetoniidae

CG Thomson, 1859

0E294E32-4D15-509E-ACFF-AA30CB116B14

##### Type species.

*Cetonia marmorata* Fabricius, 1792

#### Protaetia (Liocola) speculifera

Taxon classificationAnimaliaColeopteraCetoniidae

(Swartz, 1817)

6376EE7A-9E1F-5B80-AB6B-78FC9863A039

Fig. 8

Cetonia speculifera Swartz, 1817: 53.

##### Distribution.

In the Palearctic Region this species is only known from the Chinese provinces of Hainan, Henan, Hunan and Jiangxi (Sakai and Nagai 1998; Bezděk 2016). It has also been recorded in the Oriental Region from northern Vietnam and Laos (Krajcik 2011).

##### Material examined.

1♂: Coloane, A-Mà Cultural Village, 19 Jun 2019, dead under tree, R Perissinotto & L Clennell (MACT).

##### Other Macau records.

Alto de Coloane, 14 Jun 2019, on sap flow of *Albizia lebbeck*, R Perissinotto & L Clennell; ibidem 5 Jul 2020, on sap flow of *Sapium discolor*, R Perissinotto & L Clennell; Coloane Village, 8 Jul 2020, landing on tree trunk, R Perissinotto & L Clennell.

##### Remarks.

This species has a rather sporadic occurrence in Macau, having been observed only twice in 2019 and always on sap flows of *Albizia lebbeck*, and then again on another two occasions in 2020, on sap flows of *Sapium discolor* and in hovering flight respectively (pers. obs.). Adult size varies in the approximate range of 20–23 mm TL and 11–13 mm MW. Although it has not been recorded formally from nearby Hong Kong, a few observations reported on the citizen science platform iNaturalist from that area (e.g., https://www.inaturalist.org/observations/25994523; https://www.inaturalist.org/observations/24358351; https://www.inaturalist.org/observations/1126433) indicate that this species [or the closely related P. (L.) brevitarsis (Lewis, 1879)] may occur there too. It has probably been overlooked in the past, as superficially it resembles quite well P. (C.) orientalis orientalis both in size and general appearance. Even an alerted observer needs to be within close distance in order to be able to appreciate the stockier body shape, the reduction of white maculation on the dorsal surface and the gold-red sheen that allow the diagnosis of this species against P. (C.) orientalis orientalis. Adult activity of P. (L.) speculifera in Macau seems to be restricted to the summer months.

**Figure 8.**
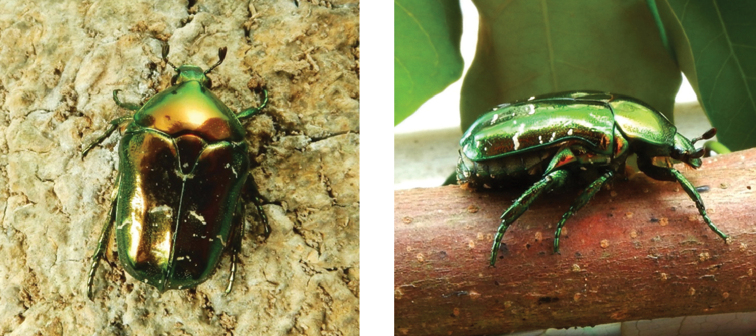
Protaetia (Liocola) speculifera (Swartz, 1817): dorsal (left) and side (right) views of typical specimens observed at Coloane on 14 Jun 2019 and 8 Jul 2020, respectively (photographs: Lynette Clennell).

#### Subgenus Potosia

Taxon classificationAnimaliaColeopteraCetoniidae

Mulsant & Rey, 1871

2CC77E4D-1ABC-592C-BFE2-AFD71C9B897B

##### Type species.

*Cetonia floricola* Herbst, 1790 (= *Cetonia metallica* Herbst, 1782)

#### Protaetia (Potosia) intricata

Taxon classificationAnimaliaColeopteraCetoniidae

WW Saunders, 1852

278F35D2-947B-5C4F-89C3-9516A146AB04

Fig. 9

Protaetia intricata WW Saunders, 1852: 31

##### Distribution.

This species appears to be rather restricted geographically, having so far been recorded only in the Chinese provinces of Fujian and Zhejiang (Bezděk 2016; Krajcik 2011).

##### Material examined.

1♂: Coloane, Ká-Hó, 2 Jun 2019, on flowers of *Syzigium buxifolium*, R Perissinotto & L Clennell (SJCP).

##### Other Macau records.

Coloane, Ká-Hó, 29 May 2020, on flowers of *Syzigium buxifolium*, R Perissinotto [identification uncertain].

##### Remarks.

This is certainly the rarest cetoniine recorded so far in Macau, having been observed with certainty only once in June 2019, feeding on flowers of *Syzigium buxifolium*. A second potential specimen was observed in a nearby locality on the same flowers in May 2020, but its identification could not be conclusively verified as it was too far above the ground. According to S. Jákl (pers. comm.), this species is extremely rare throughout its limited distribution range and to our knowledge has not been reported from Hong Kong yet. The approximate size of the 2019 male specimen was 15 mm TL and 9 mm MW.

**Figure 9.**
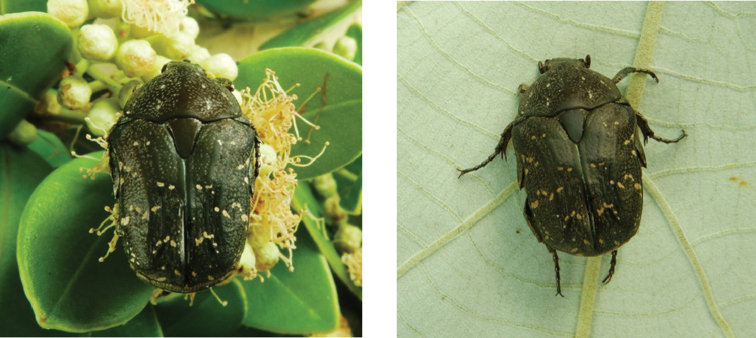
Protaetia (Potosia) intricata WW Saunders, 1852: dorsal habitus of the male specimen observed at Coloane on 2 Jun 2019 (photographs: Lynette Clennell).

#### Subgenus Protaetia

Taxon classificationAnimaliaColeopteraCetoniidae

Burmeister, 1842

AD64D9AD-A7A9-5442-96A9-09B66124ED56

##### Type species.

*Cetonia mandarina* Weber, 1801 (= *Cetonia fusca* Herbst, 1790)

#### Protaetia (Protaetia) fusca

Taxon classificationAnimaliaColeopteraCetoniidae

(Herbst, 1790)

F5D2CBC4-184A-585A-B5EC-E328992F984E

Fig. 10

Cetonia fusca Herbst, 1790: 257

##### Distribution.

Occurring widely in the Palearctic Region, especially the Chinese provinces and municipalities of Fujian, Guangdong, Guangxi, Hainan, Hubei, Jiangxi, Zhenjang, Shanghai, the Hong Kong SAR and the island of Taiwan as well as Japan and India’s Sikkim Province (Bezděk 2016). Also found in the Oriental, Australian, Afrotropical, and Pacific regions (Bezděk 2016), particularly across SE Asia, Australia, New Guinea, Madagascar, Mauritius, Hawaii, Polynesia, Micronesia, and Melanesia (Sakai and Nagai 1998; Krajcik 2011). It has recently been intercepted in Florida and in the Caribbean countries of Bahamas and Barbados (Woodruff 2006), thus becoming a near-cosmopolitan species.

##### Material examined.

1♂: Macau, University of East Asia, Jun 1990, ER Easton leg (UMEC); 1♂: Coloane, Cheoc Van, 29 Jun 2019, crushed on sidewalk, R Perissinotto & L Clennell (MACT).

##### Other Macau records.

Coloane, Hác-Sá, 4 Apr 2019, on flowers of *Ligustrum sinense*, R Perissinotto; Coloane Village, 17 Jul 2020, on building wall, L Clennell; Coloane, Oscar Farm, on rice stem, 24 Oct 2020, Kit Chang (pers. comm.).

##### Remarks.

Despite being one of the most worldwide spread cetoniine, this species is extremely scarce in Macau. Adults are active mainly in spring and summer and range in size from approximately 13 to 15 mm TL and from 8 to 9 mm MW. In Macau, they have been observed feeding only on flowers of *Ligustrum sinense*, but the widely used common name of Asian mango flower beetle for the species indicates a diet with this staple component in its natural habitat. Globally, it has actually shown a very variable diet, including a multitude of flowers, fruits and even bee honey. In Hawaii, where it was first recorded in 1949 (Maehler 1950), it is regarded as a pest, causing damage to commercially cultivated roses, maize and a wide variety of flowers and fruits. The damage caused has been regarded significant enough to justify the introduction of parasitic wasps from other regions, in an effort to exert biological control over its rapidly expanding population (Woodruff 2006). The entire life cycle of this species, including egg, 1^st^–3^rd^ instar larvae and pupa, was comprehensively described and illustrated by Simpson (1990). Larvae are compost feeders and have not been observed to attack roots of healthy plants and thus, unlike their adults, are not regarded as pests (Simpson 1990).

**Figure 10.**
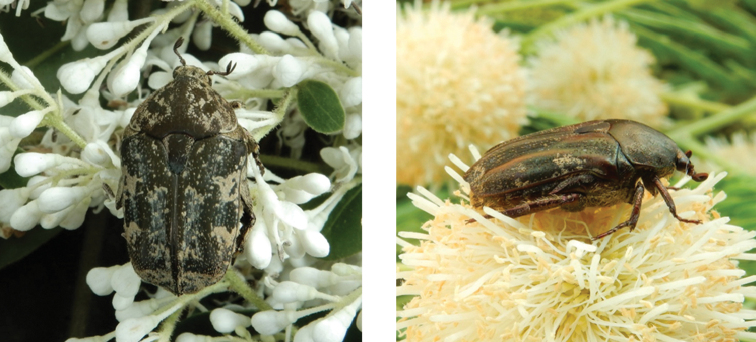
Protaetia (Protaetia) fusca (Herbst, 1790): dorsal (left) and side (right) views of typical specimens observed at Coloane on 4 Apr 2019 and 17 Jul 2020, respectively (photographs: Lynette Clennell).

### Tribe Schizorhinini Burmeister, 1842

#### Genus Agestrata

Taxon classificationAnimaliaColeopteraCetoniidae

Eschscholtz, 1829

D1A0C825-9457-5F54-AEDB-6ED77B84550D

##### Type species.

*Agestrata luconica* Eschscholtz, 1829

#### Agestrata orichalca orichalca

Taxon classificationAnimaliaColeopteraCetoniidae

(Linnaeus, 1769)

3020A26A-575B-5B66-B61A-6E8DCCA01060

Fig. 11

Scarabaeus orichalcus Linnaeus, 1769: 504.

##### Distribution.

North-eastern India (Sikkim and Arunachal Pradesh), the Chinese provinces of Guangdong, Guangxi and Hainan, the Hong Kong SAR and Taiwan. Also widespread in the Oriental Region (Bezděk 2016), occurring specifically in Myanmar, Vietnam, Laos, Thailand, Malaysia and the Indonesian islands of Sumatra and Java (Sakai and Nagai 1998; Krajcik 2011).

##### Material examined.

1♂: Macau, University of East Asia, 28 May 1989, ER Easton leg (UMEC); 1♂: Coloane, Cheoc Van, 15 Jun 2019, crushed on road under street light, R Perissinotto & L Clennell (MACT); unknown sex: Alto de Coloane, 18 Oct 2020, elytron found under spot-light, R Perissinotto & L Clennell (MACT).

##### Other Macau records.

Taipa, University of East Asia Campus, 3 Sep 1991, near library (in Easton 1991: 111, misspelt as *Agestrata orichalea*); No locality and date, 42 mm (in Pun and Batalha 1997: 65, fig. 107, misspelt as *Agestrata orichalcea*); Alto de Coloane, 18 Aug 2020, on spot-light surface, R Perissinotto & L Clennell; St. Francis Xavier’s Parish [Coloane], 18 Aug 2020, 14:55, L Clennell (https://www.inaturalist.org/observations/56913518).

##### Remarks.

This is by far the largest cetoniine beetle in Macau, reaching a total length of 40–45 mm and a maximum width of 18–20 mm. Although it is regularly recorded in nearby Hong Kong (see e.g., https://www.inaturalist.org/observations?place_id=7613&subview=grid&taxon_id=127588), it is a rare occurrence in Macau. During this study only two males were recorded, one crushed on a road under a street light and a second, which also died after flying into an incandescent spot-light at the Coloane A-Mà statue. The remnants of a third specimen were also retrieved in October 2020 under the same spot-light. According to Yiu (2010), this species feeds on a variety of fruits in captivity and is attracted to artificial light at night. The 3^rd^ instar larva of this species was comprehensively described and illustrated by Zhang (1984). The larval stage is most probably strictly saproxylic, and thereby depends on availability of decomposing tree trunks, which are rapidly disappearing in the area as more and more parts of the remaining natural vegetation are converted to city parks and gardens.

**Figure 11.**
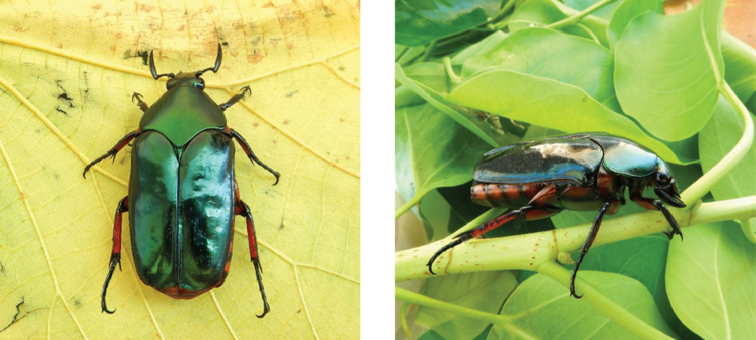
*Agestrata orichalca orichalca* (Linnaeus, 1769): dorsal (left) and side (right) views of male specimen observed at Alto de Coloane on 18 Aug 2020 (photographs: Lynette Clennell).

#### Genus Thaumastopeus

Taxon classificationAnimaliaColeopteraScarabaeidae

Kraatz, 1885

B8D93851-3445-56FB-B732-C0BB898130E2

##### Type species.

*Lomaptera mohnikii* J. Thomson, 1877

#### Thaumastopeus shangaicus

Taxon classificationAnimaliaColeopteraScarabaeidae

Neervoort van de Poll, 1886

D6D827F2-3D9C-5CA2-B190-5FA902544A61

Fig. 12

Thaumastopeus shangaicus Neervoort van de Poll, 1886: 181.

##### Distribution.

Known in the Palearctic Region from the Chinese provinces of Hainan and Yunnan, the Shanghai Municipality and the Hong Kong SAR (Yiu 2010; Bezděk 2016). Also widespread in the Oriental Region, specifically in Vietnam, Thailand, Laos, peninsular Malaysia as well as Sumatra and the Nias Islands in Indonesia (Sakai and Nagai 1998; Krajcik 2011).

##### Material examined.

1♀: Coloane Village, 31 Mar 2019, extracted prematurely from broken cocoon, R Perissinotto & L Clennell (MACT); 1♀: Coloane Village, 22 May 2020, on flowers of *Psychotria serpens*, R Perissinotto (MACT); 1♂: ibidem 28 May 2020, dead on roadside, R Perissinotto (MACT).

##### Other Macau records.

No locality and date, 23 mm [in Pun and Batalha 1997: 66, fig. 110, reported as *Thaumastopeus nigritus* (Frohlic)]; Coloane Village, 14 Jun 2019, on flowers of *Paliurus spina-christi*, R Perissinotto & L Clennell; ibidem 30 Apr 2020, on flowers of *Psychotria serpens* R Perissinotto & L Clennell; Coloane, Hác-Sá, 29 Apr 2019, emerged from cocoon found in decomposing wood, R Perissinotto & L Clennell; Coloane, Cheoc Van, 4 May 2019, on flowers of *Litsea glutinosa*, R Perissinotto & L Clennell; Alto de Coloane, 23 May 2020, R Perissinotto & L Clennell; ibidem 11 Jul 2020, on sap flow of *Sapium discolor*, R Perissinotto & L Clennell; Macau, Barra Hill, 16 Apr 2019, on flowers of *Ligustrum sinense*, R Perissinotto & L Clennell; Coloane Village, 1 Jun 2019 13:51, Hannah Leung (https://www.inaturalist.org/observations/27733112); Taipa Pequena, 23 May 2020 9:56, Eric Kwan (https://www.inaturalist.org/observations/47007347).

##### Remarks.

This is the second largest cetoniine species found in Macau, attaining a size in the range of 22–30 mm TL and 9–13 mm MW. Specimens are very stable in their colour, which is generally shiny and black with a dark blue sheen. It has been confused in the past with the closely related *T. nigritus* (Frölich, 1792) (e.g., Pun and Batalha 1997; Yiu 2010; Yiu and Yip 2011), which actually occurs mainly in the Indian subcontinent including the Himalayan region (Krajcik 2011; Bezděk 2016). The correct identification of *T. shangaicus* in the Macau and Hong Kong area has now been conclusively established through analysis of aedeagal material (S. Jákl, pers. comm.). The species is saproxylic, with larvae, cocoons and even adults having been found regularly inside decomposing tree trunks and branches (pers. obs.). Adults appear to have a very broad diet, feeding on fruits, flowers, and sap flows, but do not seem to be attracted into fruit-baited traps. In Macau, several specimens were observed on sap flows of *Sapium discolor* in July 2020 and in Hong Kong this species has often been found feeding on ripe fruits of longan and figs (Yiu 2010). Among the plants where *T. shangaicus* has been recorded feeding on flowers are *Litsea glutinosa*, *Psychotria serpens*, *Paliurus spina-christi*, *Ligustrum sinense*, and *Acronychia pedunculata*.

**Figure 12.**
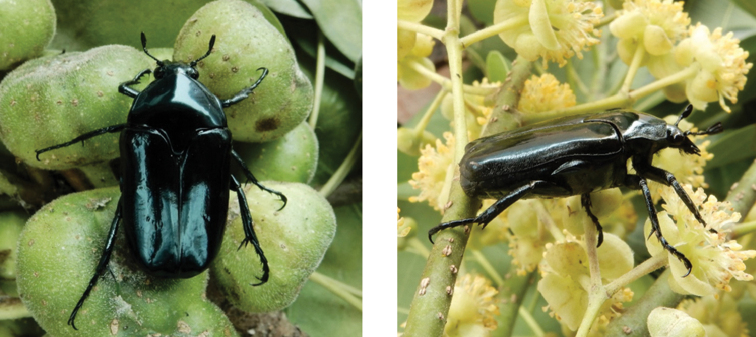
*Thaumastopeus shangaicus* Neervoort van de Poll, 1886: dorsal (left) and side (right) views of typical specimens observed at Coloane on 30 Apr 2019 and 23 May 2020, respectively (photographs: Lynette Clennell).

### Tribe Taenioderini Mikšić, 1976

#### Genus Euselates

Taxon classificationAnimaliaColeopteraCetoniidae

J Thomson, 1880

5AA76D5F-EDF1-535A-B3E0-D90DC7973426

##### Type species.

Euselates magna J Thomson, 1880

#### Subgenus Euselates

Taxon classificationAnimaliaColeopteraCetoniidae

J Thomson, 1880

855B88D1-3168-5C63-B318-7FFC015D2B81

##### Type species.

*Euselates magna* J Thomson, 1880

#### Euselates (Euselates) magna

Taxon classificationAnimaliaColeopteraCetoniidae

J Thomson, 1880

A55004E5-8C93-53CD-A26A-332D6E806AB7

Fig. 13

Euselates magna J. Thomson, 1880: 277

##### Distribution.

Known in the Palearctic Region from the Chinese provinces of Hainan, Hubei and the Hong Kong SAR (Bezděk 2016). It is also widespread in the Oriental Region specifically in Vietnam, Laos and Thailand (Sakai and Nagai 1998; Krajcik 2011).

##### Material examined.

1♀: Coloane, Hác-Sá, 14 May 2019, on flowers of *Psychotria serpens*, R Perissinotto (MACT); 1♂, 1♀: Coloane Village, 2 Jul 2019, dead on roadside, R Perissinotto & L Clennell (MACT).

##### Other Macau records.

Coloane, Hác-Sá, 28 Apr 2019, on flowers of *Lonicera japonica*, R Perissinotto & L Clennell; ibidem 3 May 2019, on flowers of *Psychotria serpens*, R Perissinotto & L Clennell; ibidem 15 May 2020, R Perissinotto; St. Francis Xavier’s Parish [Coloane], 16 May 2020 10:35, L Clennell (https://www.inaturalist.org/observations/56121519); ibidem 11 Jul 2020 9:45, Kisu Wong (https://www.inaturalist.org/observations/57338916); ibidem 11 Jul 2020 10:45, Kit Chang (https://www.inaturalist.org/observations/52662946).

##### Remarks.

This species has been previously reported from nearby Hong Kong with its synonymic name of *E. schoenfeldti* Kraatz, 1893 (Yiu 2010; Yiu and Yip 2011). In Macau it is occasionally seen between late April and August, but only in the largest patches of natural vegetation. Adults exhibit an approximate size in the range of 19–22 mm TL and 8–10 mm MW. They are very consistent in their colour pattern and are active during the hottest part of the day, but even under overcast conditions. They are typically flower feeders, having been observed on *Litsea glutinosa*, *Lonicera japonica*, *Psychotria serpens* and in Hong Kong also on *Cleistocalyx operculatus* (Yiu 2010). On one occasion, a male specimen was found inside a fruit-baited trap containing a mixture of fermenting banana, pineapple, brown sugar, and red wine. The larvae are unknown, but most likely saproxylic, as on two occasions females were observed while entering crevices in decomposing tree trunks.

**Figure 13.**
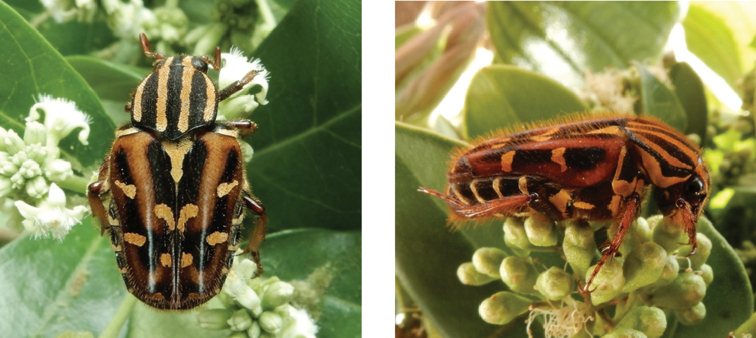
Euselates (Euselates) magna J Thomson, 1880: dorsal (left) and side (right) views of typical specimens observed at Coloane on 3 May 2019 and 15 May 2020, respectively (photographs: Lynette Clennell).

## Discussion

Results of this census show a significant increase in the number of cetoniine species occurring in the Macau SAR to eleven, compared to only four reported in previous publications (Easton 1991; Easton 1993; Pun and Batalha 1997). This is undoubtedly related to the escalation in observation efforts applied in this study, with visits in the field undertaken almost on a daily basis for a period of more than two years and covering virtually all the major pockets of natural vegetation that are still found in the region. Thus, the total number of cetoniine species recorded in Macau now compares relatively well with that of Hong Kong, where 15 species have so far been confirmed (Yiu 2010; Bezděk 2016). This is particularly relevant, considering that the total surface area of the Hong Kong SAR is approximately 36 times larger than that of Macao and exhibits a much larger diversity of vegetation types and habitats (Dudgeon and Corlett 1994). For a larger-scale regional comparison, it is worth noting that the cetoniine diversity observed in Macau and Hong Kong is also similar to that recorded so far in the mainland province of Guangdong (17 species), but drastically lower than the numbers recorded on the islands of Hainan (36 species) and especially Taiwan (80 species).

Of special interest are the two *Protaetia* species [P. (L.) speculifera and P. (P.) intricata] that were previously unreported from the region, including Hong Kong, possibly due to their low frequency of occurrence in this area or to their superficial resemblance with other sympatric species. Both factors appear to be involved, as the two species were observed only on two occasions and only once, respectively, in each year of the census. Protaetia (P.) intricata is known for its remarkable scarcity across its entire and relatively small distribution range (S. Jákl, pers. comm.). Because of its small size and dark brown to olive-green background colour, it can potentially be confused with poorly ornamented varieties of the more common P. (P.) fusca by an untrained eye, or when not inspected at close range. Protaetia (L.) speculifera, on the other hand, is regarded as relatively common and much more widely distributed than P. (P.) intricata. However, it can be easily confused with the numerically dominant P. (C.) o. orientalis, and ever more so with the closely related P. (L.) brevitarsis, when not analysed in detail. The latter species has apparently been recorded in the mainland province of Guangdong, but not in either Macau or Hong Kong (Bezděk 2016). Recent postings from Hong Kong in the citizen science platform iNaturalist, however, show several specimens with reduced dorsal white maculation and dark green background colour or reddish gold sheen. These are consistent with the superficial appearance of P. (L.) brevitarsis and P. (L.) speculifera, respectively, and it is thus likely that at least one of the two species, or perhaps even both, may occur there. Further detailed analyses of some of these specimens will be required in order to test this hypothesis, and this will have to include a comparison of aedeagal parameres.

While the cetoniine diversity of Macau is larger than expected, what is of concern is the relatively poor abundance of most species recorded. Indeed, the frequency of occurrence of the various species reveals that only P. (C.) o. orientalis and Glycyphana (G.) laotica can be regarded as widespread and seasonally relatively common in Macau. All the others were recorded only on a few occasions and generally as single individuals, which is an indication that most local populations are under extreme stress and on the verge of becoming unsustainable. Some of the records may actually represent migrants from neighbouring regions on a dispersal flight and, thus, may not even have viable populations established within the Macau SAR.

Unfortunately, the few remaining areas of natural vegetation in the territory, mainly hills, are being systematically manipulated with undergrowth vegetation and dead or moribund trees removed, shredded and turned to compost. This process was escalated in the wake of the destructive impact of Typhoon Hato in August 2017, when trees were uprooted and damaged on a large-scale. The prompt intervention of the authorities ensured that all the damaged trees were cut and removed and, in their place, new young trees were planted within an ongoing rehabilitation programme. The problem is that these new trees are planted in an plantation-type manner, with ample space between each other and removal of any undergrowth inadvertently regarded as weeds. Trees are also regularly pruned of their lower branches. While all this is presumably done with the purpose of improving the aesthetic appearance of these green areas, it deprives the soil of its natural buffer against desiccation and extreme temperatures, thereby annihilating the habitat of soil invertebrates, including cetoniine larvae. The removal and destruction of the older, dead, or moribund trees also deprives the larval stages of all saproxylic species of their natural habitat. This seems to be impacting negatively in particular the two Schizorhinini species, which are also the largest cetoniines occurring in Macau, namely *Agestrata orichalca* and *Thaumastopeus shangaicus*. Both were regularly recorded in the past (Easton 1991; Pun and Batalha 1997), but now appear to have become a rare occurrence and are possibly under serious threat because of the rapid disappearance of their habitat, which consists of large decomposing tree trunks and branches that are still standing (pers. obs.). Thus, should the practices highlighted above continue into the future, it seems inevitable that invertebrate biodiversity in the SAR will steadily decline, with some species probably becoming locally extinct, including fruit and flower chafers.

## Supplementary Material

XML Treatment for Gametis

XML Treatment for Gametis bealiae

XML Treatment for Gametis jucunda

XML Treatment for Glycyphana

XML Treatment for Glycyphana (Glycyphana) horsfieldii

XML Treatment for  Glycyphaniola

XML Treatment for Glycyphana (Glycyphaniola) laotica

XML Treatment for Protaetia

XML Treatment for  Calopotosia

XML Treatment for Protaetia (Calopotosia) orientalis orientalis

XML Treatment for Liocola

XML Treatment for Protaetia (Liocola) speculifera

XML Treatment for Potosia

XML Treatment for Protaetia (Potosia) intricata

XML Treatment for Protaetia

XML Treatment for Protaetia (Protaetia) fusca

XML Treatment for Agestrata

XML Treatment for Agestrata orichalca orichalca

XML Treatment for Thaumastopeus

XML Treatment for Thaumastopeus shangaicus

XML Treatment for Euselates

XML Treatment for Euselates

XML Treatment for Euselates (Euselates) magna
